# NeoHebbian synapses to accelerate online training of neuromorphic hardware

**DOI:** 10.1038/s41598-026-35641-z

**Published:** 2026-02-18

**Authors:** S. Pande, S. S. Bezugam, T. Bhattacharya, E. Wlazlak, A. Chakravorty, B. Chakrabarti, D. Strukov

**Affiliations:** 1 Indian Institute of Technology Madras, Chennai, Tamil Nadu 600036 India; 2 UC Santa Barbara, Santa Barbara, CA 93106-9560 USA

**Keywords:** BPTT, e-prop, NeoHebbian synapses, Online learning, Reinforcement learning, Recurrent spiking neural network, ReRAM, Thermal synapses, Three-factor learning rule, Electrical and electronic engineering, Computational science

## Abstract

Neuromorphic systems that employ advanced synaptic learning rules, such as the three-factor learning rule, require synaptic devices of increased complexity. Herein, a novel neoHebbian artificial synapse utilizing ReRAM devices has been proposed and experimentally validated to meet this demand. This synapse features two distinct state variables: a neuron coupling weight and an “eligibility trace” that dictates synaptic weight updates. The coupling weight is encoded in the ReRAM conductance, while the “eligibility trace” is encoded in the local temperature of the ReRAM and is modulated by applying voltage pulses to a physically co-located resistive heating element. The utility of the proposed synapse has been investigated using two representative tasks: first, temporal signal classification using Recurrent Spiking Neural Networks (RSNNs) employing the e-prop algorithm, and second, Reinforcement Learning (RL) for path planning tasks in feedforward networks using a modified version of the same learning rule. System-level simulations, accounting for various device and system-level non-idealities, confirm that these synapses offer a robust solution for the fast, compact, and energy-efficient implementation of advanced learning rules in neuromorphic hardware.

## Introduction

Emulating the biophysical dynamics of the brain by manipulating naturally available physical dynamics lies at the core of neuromorphic computing and is what holds the key to achieving at-par energy efficiency and cognitive capabilities of the human brain^1–5^. Realizing the full potential of neuromorphic computing requires the development of a computational paradigm that reasonably mimics the structure and functionality of the brain at various levels of abstraction while also being conducive to efficient hardware implementation using state-of-the-art technologies^6–9^.The latter can be achieved using memristive devices, which are known for emulating the synaptic functionality due to their ability to tune the conductance to an arbitrary value within its physical dynamic range^10–13^. Additionally, when arranged in crossbar arrays, these devices enable area and energy-efficient in-memory computing by offering massive parallelism. Several studies have reported chip-level demonstrations of neural network accelerators using various memristive devices^14–17^. The former is achieved by adopting spiking neural networks (SNNs). SNNs are known for offering the brain-inspired computational paradigm that comprises approximated neuro-inspired neuron models as activation functions interconnected with synaptic weights and transmit information using asynchronous spike-based events^18–21^. Thus, using memristor-based hardware to implement SNNs is a compelling alternative for attaining energy efficiency and cognitive performance comparable to those of a biological brain.

SNNs can be trained using the Hebbian learning rules, such as spike-timing-dependent plasticity (STDP) or its variants^22–24^. In STDP-based learning, the timing and sequence of pre- and post-synaptic spikes determine the magnitude and direction of weight changes^25^. Several experimental demonstrations have shown that SNNs trained with the STDP algorithm can learn to detect temporal correlations within spike trains in an unsupervised manner^26–28^. Additionally, large-scale experimental demonstrations have investigated the potential benefits of SNNs^29–34^. Despite their biological plausibility, SNNs trained using STDP perform poorly on relatively complex tasks primarily due to their focus on local optimization and lack of a global error signal, as seen in artificial neural networks (ANNs) trained with backpropagation^35^. As a result, the performance of spike-based learning algorithms has often been overshadowed by gradient-based methods used in non-spiking networks. Another significant limitation of SNNs trained with Hebbian learning rules is their inability to model tasks involving long-term temporal dependencies^36^. While recurrent spiking neural networks (RSNNs) offer a potential solution for modeling such tasks, their training algorithms struggle to assign importance to past neural states for errors observed in the present, making it difficult to determine the necessary adjustments to the network’s learnable parameters to achieve desired performance^36,37^. This issue, known as the temporal credit assignment problem, is not unique to RSNNs but also exists in ANNs. ANNs address this problem using the backpropagation through time (BPTT) algorithm^38^. However, BPTT requires unfolding the network and propagating errors backward through time^38,39^, which, while effective for modeling long-term temporal sequences, demands extensive memory, high training time, and significant computational resources, thereby limiting its use in neuromorphic hardware^40^.

The eligibility propagation (e-prop) algorithm effectively addresses the temporal credit assignment problem in a biologically plausible way^36^. Studies have shown that RSNNs trained with e-prop algorithms can learn online and handle complex tasks efficiently^36^. The e-prop algorithm is a special case of the three-factor learning rule, where synaptic plasticity is influenced not only by the pre-synaptic and post-synaptic neuron signals (as in standard Hebbian learning) but also by an additional third signal. Typically, a three-factor learning rule for synaptic plasticity can be expressed as^35^:

$$\begin{aligned} \dot{w} = F(M, pre, post). \end{aligned}$$

In this equation, $\dot{w}$ represents the rate of change of the synaptic weight. The variable *M* denotes the third signal, and the function *F* defines the specific learning rule. Three-factor learning rules, including their variants, tackle the issues associated with SNNs by introducing local eligibility traces. These traces, combined with the coupling weight, maintain a fading memory of pre-synaptic activity. Additionally, they make the global error signal (the third signal) locally accessible at the synapse, along with the pre-and post-synaptic signals, facilitating local learning. In the context of temporal modeling tasks, these characteristics eliminate the need for the network to unfold and propagate backward in time, resulting in substantial savings in computational resources and accelerating the training process of neuromorphic hardware.

This work focuses on developing a synaptic element tailored for hardware implementation of the e-prop learning algorithm. Our key contributions are as follows: (1) We propose a novel artificial synapse with a two-terminal heater 3D-integrated with a ReRAM cell. This design utilizes intentionally introduced self-thermal coupling between the heater and ReRAM to encode the eligibility trace through the local temperature of the ReRAM, while the non-volatile conductance levels represent the synaptic weights. (2) We provide a comprehensive analysis of the proposed synapse’s operation, including its physical mechanisms and various non-idealities. The core operating principle is experimentally validated, and its implementation at the array level is studied within the context of the e-prop algorithm. (3) We present a numerical model to further investigate the synapse’s operation and assess its scalability. (4) We evaluate the synapse’s performance on two representative tasks using hardware-aware network simulations, accounting for device- and array-level non-idealities.

The remainder of the manuscript is organized in the following order: section “Eligibiility-based learning” presents a high-level description of eligibility-based learning, followed by a discussion on the proposed synapse operation, related experimental results, unit cell design, and its array-level operation in the section “Thermal NeoHebbian synapse”. The section “Numerical modeling covers the numerical modeling of the proposed synapse. The section “Benchmark simulations” details system-level simulations used to benchmark the performance benefits of the proposed synapse on two representative tasks: reinforcement learning in SNNs and the more complex TIMIT phoneme classification task in RSNNs. Finally, we conclude by summarizing the scope and limitations of the proposed synapse in the section “Discussion & summary”.

## Eligibiility-based learning

**Fig. 1:**
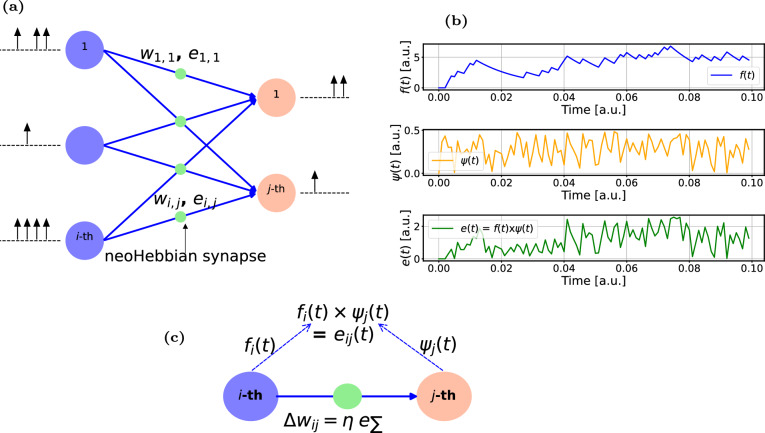
**a** Schematic of a spiking neural network incorporating neoHebbian synapses. **b** The evolution of signals *f*(*t*), $\psi (t)$, and *e*(*t*) during the dataframe presentation. $f_{i}(t)$ and $\psi _{j}(t)$ represent signals from the $i-$th pre-synaptic neuron and $j-$th post-synaptic neuron, respectively. $e_{ij}(t)$ is obtained by multiplying $f_{i}(t)$ and $\psi _{j}(t)$. **c** Characteristic features of a neoHebbian synapse - computing $e_{ij}(t)$ and accumulating it (i.e., e_Σ_) during the data frame presentation. During the weight update, the weight change ($\Delta w_{ij}$) is proportional to the accumulated *e*(*t*). $\eta$ represents the learning rate.

A high-level description of eligibility-based learning in SNNs utilizing neoHebbian synapses is discussed in this section. Figure 1a shows a schematic of the SNN where input neurons are connected to output neurons using neoHebbian synapses. NeoHebbian synapses exhibit both short-term dynamics and long-term plasticity, characterized by the synaptic “eligibility trace” ($e$) and coupling weight ($w$), respectively. The neuronal firing activity at the pre- and post-synaptic neurons dictates the updates in eligibility trace values. These traces serve as temporal markers that record the past activities of the synapse. When the synaptic weights are to be updated, eligibility traces interact with neuromodulator signals to determine the extent and direction (increase or decrease) of synaptic weight changes. In other words, the eligibility trace serves as an additional gating signal that, in conjunction with pre- and post-synaptic activities, influences long-term plasticity and is regulated by the common (two-factor) Hebbian rule.

The computation of the eligibility state ($f(t)$) takes place at the pre-synaptic neuron, while the pseudo-gradient ($\psi (t)$) is generated at the post-synaptic neuron. The signal $f(t)$ acts as a low-pass filtered version of the incoming spike train, giving the synapse a fading memory of recent pre-synaptic activity. The role of $\psi (t)$ is different: it serves as a surrogate derivative for the spiking nonlinearity. Because the spike-generation function is a hard threshold, its true derivative is either zero or undefined and therefore not useful for learning. To overcome this, $\psi (t)$ is defined as a smooth approximation that is non-zero only within a narrow window around the firing threshold. Importantly, this pseudo-derivative window is always non-negative and acts only as a scaling factor, while the sign of $\psi (t)$ is provided by the global learning signal $L(t)$. In practice, this means that whenever the membrane potential comes close to threshold, the neuron is assigned a non-zero gradient of the appropriate sign to enable effective weight updates during learning. Together, $f(t)$ and $\psi (t)$ form the eligibility trace $e(t) = f(t)\times \psi (t)$, which captures both the pre-synaptic history and the post-synaptic sensitivity to its membrane potential. The training process operates in batches, where data within each batch, termed as a dataframe, is sequentially processed over $U$ time steps. During the training process, $e(t)$ is computed and accumulated over the presentation of the dataframe. Figure 1b shows the evolution of signals $f(t)$, $\psi (t)$, and $e(t)$ during the dataframe presentation. Subsequently, at the end of the dataframe presentation, the coupling weights ($w_{ij}$) are updated proportionally to e_Σ_, where e_Σ_ denotes the accumulated $e(t)$ over the dataframe presentation. Overall, the eligibility-based learning approach allows the network to associate specific spike timings with subsequent rewards or punishments, enhancing its ability to perform tasks that require temporal linking of events, such as sequence learning and reinforcement learning, where outcomes are delayed from actions. Detailed equations related to eligibility-based learning are provided in the supplementary information (Section 1) and the section “Benchmark simulations” in the main manuscript.

**Fig. 2:**
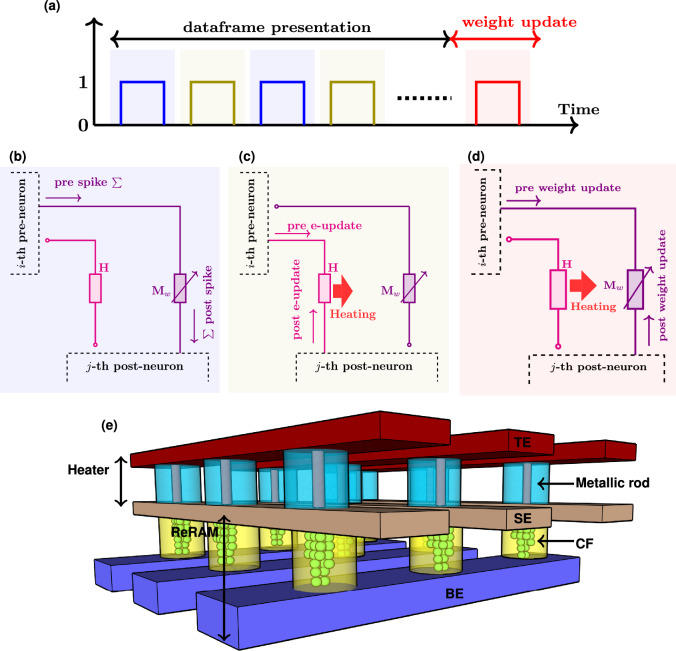
High-level description of the thermal neoHebbian synapse operation: **a** Three key stages involved in the training operation of e-prop: spike integration (shaded blue), eligibility-update (shaded olive), and weight update (shaded red). Arrangement of the heater and ReRAM during **b** Spike integration, **c** e-update, and **d** Weight update phases. The red arrow depicts the thermal coupling between the heater and ReRAM. **e** Design of a crossbar array illustrating 3D-integrated heater and ReRAM cells.

## Thermal NeoHebbian synapse

The high-level functionality of the proposed synapse in the context of the e-prop training algorithm is outlined in the following. The training process in e-prop consists of three key stages: the spike integration (or inference) phase, the eligibility update (e-update) phase, and the weight update phase. The spike integration and e-update occur during the data frame presentation, while the weight update is executed after the data frame presentation (refer to Fig. 2a). Consider the heater (*H*) and ReRAM ($M_{w}$) arrangement as shown in Fig. 2b. In this, $M_{w}$ acts as coupling weight and transmits weighted current spikes from *i*-th pre-neuron to *j*-th post-neuron during the spike integration phase, as shown in Fig. 2b. During the e-update phase, the heater receives appropriate signals such as *f*(*t*) and $\psi (t)$ from pre- and post-neurons, respectively, resulting in the rise in heater temperature due to Joule heating. Due to the thermal coupling between the heater and ReRAM, the local temperature of ReRAM increases proportionally to the dissipated power. The e-update phase is depicted in Fig. 2c. These operations are repeated at every step during the dataframe presentation in the training process. Finally, at the end of the data frame presentation, the accumulated temperature rise in ReRAM represents “e_Σ_”, thus satisfying the requirement of computing and storing the eligibility trace at the synapse. Subsequently, during the weight update, a fixed-amplitude programming pulse is applied, which induces a conductance change ($\Delta G_{w}$) proportional to the accumulated temperature rise (e_Σ_). The weight update phase is depicted in Fig. 2d. Essentially, the temperature-dependent switching behavior of the ReRAM is exploited to update the weights proportional to e_Σ_.

To implement the characteristic features highlighted by the high-level functionality of the thermal neoHebbian synapse, we propose the integration of a two-terminal heater cell with the ReRAM device, as depicted in Fig. 2e. The heater element comprises an insulating layer sandwiched between two metallic layers: the top electrode (TE) and the shared electrode (SE). A metallic nanorod connects the TE and SE. Upon applying a voltage between the TE and SE, a substantial current flows through the metallic nanorod, which has a high electrical conductivity, resulting in localized Joule heating. Due to the high thermal conductivity of the SE, strong thermal coupling is established between the heater element and the ReRAM. The ReRAM switching layer is sandwiched between the SE and the bottom electrode (BE). To mitigate lateral heat diffusion to adjacent cells, the nanorod structure is surrounded by an electrically and thermally insulating layer. The desired properties of the heater are akin to those used in mushroom-type phase-change memory technologies^41–43^. Consequently, suitable materials for the electrode layers include W, TiN, and TaN, and for the insulating layers, materials such as $\hbox {SiO}_{2}$, $\hbox {HfO}_{2}$, and $\hbox {TiO}_{2}$. This design minimizes area footprint by 3D integration of the heater and the ReRAM cells. The decay of the eligibility trace in this design is inherently linked to thermal properties defined at fabrication and is governed by the thermal time constant $\tau _{\textrm{TH}} = R_{\textrm{TH}}C_{\textrm{TH}}$, where $R_{\textrm{TH}}$ is the effective thermal resistance and $C_{\textrm{TH}}$ is the thermal capacitance of the heated volume. Although intrinsic thermal diffusivity is a fixed material property, the effective time constant can still be tuned through geometry and stack design. Parameters such as oxide/electrode thickness, device area, crossbar pitch, and the introduction of thermal barrier layers offer practical control knobs^44^. Details about the fabricated ReRAM layer stack and its electrical characteristics are presented in the following section.

### Experimental results

**Fig. 3:**
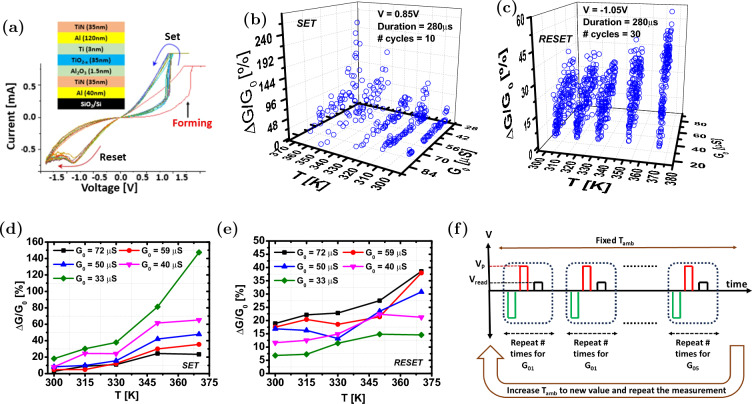
**a** Representative I-V curves measured with quasi-static DC voltage sweep at 1V/s on $250\times 250\hbox {nm}^2$ area devices. The inset provides the device stack details. Normalized percentage conductance change as a function of the initial conductance and ambient temperature is shown for the **b** SET and **c** RESET processes. The average normalized percentage conductance change as a function of ambient temperature for the SET and RESET processes is presented in (**d**) and (**e**), respectively. **f** Illustration of the measurement protocol used to obtain the data is shown in (**b**) and (**c**). The green pulse depicts the multiple SET, RESET, and read pulses required to reprogram the device to the same $G_\textrm{0}$. $V_\textrm{P}$ and $V_\textrm{read}$, respectively, denotes fixed programming pulse used to measure $\Delta G$ and read pulse.

Metal oxide memristors were fabricated using a similar process to our previous work^10^, which involved etch-down processes, and UV lithography for patterning, DC-mode magnetron sputtering for electrode deposition, and thermal annealing in forming gas to adjust non-stoichiometry. However, in this work, an oxide bilayer stack was formed using ALD to simplify the fabrication process and mitigate issues related to thickness and composition variations in the sputtering targets. Figure 3a shows typical *I*-*V* switching curves from these devices, with the inset providing details of the ReRAM layer stack. These devices exhibit low forming voltages ($\sim$2V), switching voltages ($\sim$1V), and an on/off ratio of $\sim$20 at a read voltage of 0.1V.

We now examine the temperature-dependent switching characteristics within the context of thermal neo-Hebbian synapse operation. In the proposed design, the heater and ReRAM cells are 3D-integrated, with the structure optimized to maximize thermal coupling between them. The close proximity of the heater and ReRAM, along with the high thermal conductivity of SE, allows for simplification of experimental measurements by emulating the heater’s role through modulation of the ambient temperature. Our investigation focuses on understanding the normalized conductance change $(\Delta G/G_{0})$ induced by a fixed voltage pulse as a function of the initial programmed conductance ($G_{0}$) and ambient temperature (*T*). Figure 3b and c present $(\Delta G/G_{0})$ as a function of $G_{0}$ and *T* for the SET and RESET processes, respectively.

The measurement protocol employed during the experiments is as follows: First, the ReRAM is programmed to a target initial conductance $G_{0}$ with 5% tuning accuracy, using the tuning algorithm described in^45^. Next, a programming pulse of fixed amplitude and duration is applied, followed by a read pulse to measure the conductance change ($\Delta G$). The device is then reprogrammed to the same $G_{0}$, and the measurement is repeated multiple times to collect several data points for each $G_{0}$. This procedure is repeated for all specified $G_{0}$ values. Afterward, the ambient temperature increases and the entire process is repeated. Figure 3f illustrates the measurement protocol. For the SET process, data were obtained using a pulse with an amplitude of 0.85V and a duration of 280 $\upmu$s, with measurements repeated 10 times for each combination of $G_{0}$ and *T*. For the RESET process, a pulse of - 1.05V with a duration of 280$\upmu$s was applied, and measurements were repeated 30 times for each combination of $G_{0}$ and ambient temperature. Figure 3d shows the average normalized conductance change for the SET process, where each data point represents the average of 10 measurements, while Fig. 3e shows the corresponding average values for the RESET process, with each data point representing the average of 30 measurements.

It was observed that during the SET process, the conductance change reaches its maximum when $G_0$ is close to the device’s lowest conductance state and its minimum when $G_0$ approaches the highest conductance state. Conversely, during the RESET process, the conductance change is at its maximum when $G_0$ is near the highest conductance state and at its minimum when $G_0$ is close to the lowest conductance state. This asymmetry can be explained by the underlying filament dynamics: during RESET, rupture of the conductive filament is aided by the larger current that flows when the device is in a higher conductance state. The resulting Joule heating raises the filament temperature, accelerating oxygen vacancy redistribution. As the conductance decreases, the current drops correspondingly, so the available heating becomes insufficient to further rupture the already narrow filament, leading to saturation near the low-conductance end. In the SET process, when the device is in a high-resistance state, most of the applied bias drops across the active region, resulting in a stronger electric field that drives vacancy drift and filament formation. Consequently, conductance increase is high at low conductance, but saturates as the filament thickens and the local field intensity decreases^46,47^. Overall, this behavior is attributed to the fixed dynamic conductance range in ReRAM devices, which naturally leads to saturation of conductance updates as the device approaches either extreme of its conductance window.

### NeoHebbian synapses: unit cell and array level operation

**Fig. 4:**
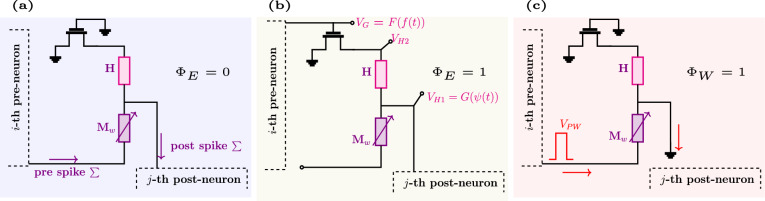
1*T*-1*H*-1*M* unit cell implementation of the thermal neoHebbian synapse. During the dataframe presentation, the operation of the synapse is time multiplexed between **a** Spike integration - $\phi _E$ = 0 and **b** e-update - $\phi _E$ = 1 phase **c** Weight update is performed at the end of the dataframe - $\phi _W$ = 1. The appropriate biasing conditions for each phase are shown in the schematic. *F*(.) & *G*(.) are defined in Eq. (2).

The unit-cell implementation of thermal neo-Hebbian synapses is shown in Fig. 4. This unit cell consists of one transistor, one heater, and one ReRAM device, hence referred to as the 1*T*-1*H*-1*M* configuration. During the spike integration phase ($\phi _E = 0$), the memristor serves as a coupling weight, facilitating the transmission of weighted current spikes from the pre-neuron to the post-neuron, while the transistor remains off, as depicted in Fig. 4a.

In the e-update phase ($\phi _E = 1$), the eligibility state $f(t)$ and the pseudo-gradient $\psi (t)$, calculated at the pre-and post-synaptic neurons, respectively, are applied across the heater. Simultaneously, the memristor ($M_w$) is decoupled from the pre-neuron, as illustrated in Fig. 4b. The applied voltage signals result in Joule heating, raising the temperature of the heater, which subsequently increases the local temperature of the ReRAM through thermal coupling. The total eligibility contribution to the weight update is stored as the cumulative temperature rise in the ReRAM during data presentation. In the final weight update phase ($\phi _W = 1$), a fixed-amplitude programming pulse is applied, leading to a change in conductance $\Delta G$, which is proportional to the accumulated eligibility trace (e_Σ_), as shown in Fig. 4c. Shared usage of one of the electrode terminals between the heater and the memristor in a 1*T*-1*H*-1*M* configuration requires time-division multiplexing between the $\phi _E$ = 0 and $\phi _E$ = 1 phases. However, introducing an additional transistor in the synaptic cell can eliminate the need for time-division multiplexing between the spike integration and the e-update phases in the 1*T*-1*H*-1*M* design, albeit with the trade-off of reduced density^48^.

An essential feature of the noeHebbian synapse is the local computation and storage of *e*(*t*). As illustrated in Fig. 1c, during e-prop operation, *e*(*t*) is computed as the product of *f*(*t*) and $\psi (t)$. In the context of the thermal neoHebbian synapse, this implies that the local temperature of $M_{w}$ should increase proportionally to the product of voltage signals *f*(*t*) and $\psi (t)$. The 1*T*-1*H*-1*M* unit cell allows local computation and storage of *e*(*t*) through appropriate biasing and voltage scaling.

During $\phi _E$ = 1, assuming the transistor is operating in the triode regime, the drain current ($I_\textrm{D}$) is given by,

$$\begin{aligned} I_\textrm{D}(t)=k\left( V_\textrm{GS}(t)-V_\textrm{TH}\right) V_\textrm{H2}(t). \end{aligned}$$

The voltage drop across the heater can be expressed in terms of the drain current as:

$$\begin{aligned} V_\textrm{H1}(t) - V_\textrm{H2}(t) = I_\textrm{D}(t) R. \end{aligned}$$

Here, *R* denotes heater electrical resistance, and *k* is a transistor-related parameter. Consequently, the power dissipated across the heater is expressed as:

1 $$\begin{aligned} P_\textrm{H}=\frac{1}{R} V_\textrm{H1}^2 \frac{\left( Rk V_{\textrm{OV}}\right) ^2}{\left( 1+Rk V_{\textrm{OV}}\right) ^2} \,. \end{aligned}$$

Now, $V_\textrm{H1}$ and $V_{\textrm{OV}}$ are scaled as follows,

2 $$V_{{{\mathrm{H1}}}} \propto \sqrt {\psi (t)} \;\& \;\;V_{{{\mathrm{OV}}}} \propto \frac{{\sqrt {f(t)} }}{{Rk(1 - \sqrt {f(t)} )}}.{\text{ }}$$

$V_{\textrm{OV}}$ denotes transistor overdrive voltage. This voltage scaling performed at the neuron site ensures that the dissipated power across the heater and, consequently, the temperature rise at the heater follows the desired proportionality:

3 $$\begin{aligned} P_\textrm{H} \propto \psi (t) \times f(t) \quad \text {;} \quad \therefore \; \Delta T \propto \psi (t) \times f(t). \end{aligned}$$

Due to thermal coupling, the power dissipated across $M_\textrm{w}$ is proportional to $P_\textrm{H}$; consequently, the local temperature of $M_\textrm{w}$ increases in relation to the product of $\psi (t)$ and *f*(*t*). More details on the related equations are provided in the supplementary information. For completeness, we note that both the pre-synaptic low-pass state *f*(*t*) and the scaled pseudo-gradient $\psi (t)$ can be mapped onto standard neuromorphic circuit primitives. In practice, *f*(*t*) can be implemented as a simple leaky integrator (e.g., RC or switched-capacitor), while $\psi (t)$ can be generated by a comparator-based or piecewise-linear window around the firing threshold and scaled by the global learning signal *L*(*t*)^49,50^.

**Fig. 5:**
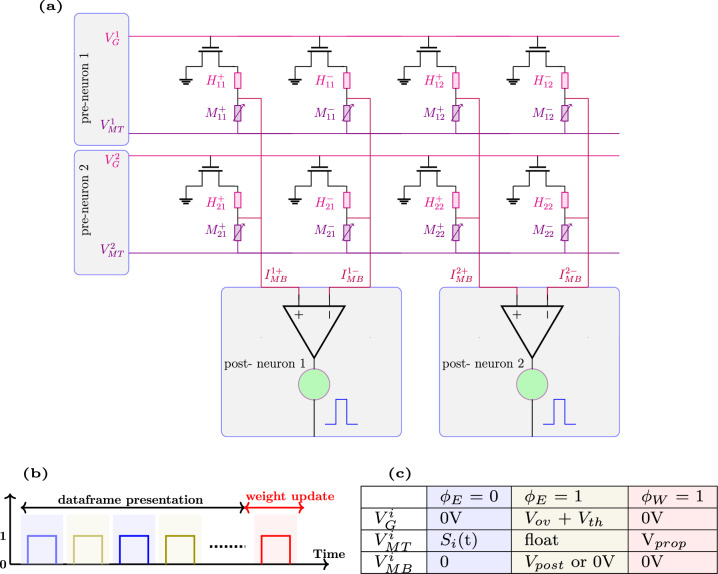
**a** Differential mode crossbar-array implementation of the 1*T*-1*H*-1*M* design. **b** Key stages in the operation of e-prop: spike integration ($\phi _E$ = 0), e-update ($\phi _E$ = 1), and weight update ($\phi _W$ = 0). **c** Biasing condition at the respective terminal during these phases.

We now discuss the array-level operation of the proposed synapse. The array-level implementation of the 1*T*-1*H*-1*M* design involves synaptic cells arranged in a differential configuration, as shown in Fig. 5a. The key stages in the operation of e-prop are shown in Fig. 5b. In this setup, two sets of synapses are utilized. The net synaptic conductance is given by $G = G^+ - G^-$, where $G^+$ denotes the total conductance of memristor $M^{+}_{w}$, and $G^-$ represents the total conductance of memristor $M^{-}_{w}$. The net conductance *G* can be increased (decreased) by potentiating (depressing) $G^+$ or depressing (potentiating) $G^-$^51,52^.

Figure 5c shows the voltage bias applied at respective terminals during spike integration, e-update, and weight update. The e-update and subsequent weight update operation in the differential mode operate as follows: During e-prop operation, *f*(*t*) maintains a strictly positive value, while $\psi (t)$ can be either positive or negative. Depending on the sign of $\psi (t)$, the e-update operation is directed towards either heater $H^{+}$ or heater $H^{-}$. For instance, when $\psi (t) > 0$, *f*(*t*) and $\psi (t)$ are applied to the terminals of heater $H^{+}$, as illustrated in Fig. 4b. Conversely, if $\psi (t) < 0$, the update is directed towards heater $H^{-}$. During the $\phi _W$ = 1 phase, a fixed amplitude programming pulse is simultaneously applied to both memristors $G^{+}$ and $G^{-}$. Consequently, the resulting change in conductances, denoted as $\Delta G^{+}$ and $\Delta G^{-}$, is directly proportional to the local temperature increase at the respective synapse during the e-update phase. Therefore, the net change in conductance ($\Delta G$) is calculated as $\Delta G = \Delta G^{+} - \Delta G^{-}$. It’s important to note that the voltage pulse used during $\phi _W$ = 1 induces insignificant conductance change if the local temperature rise at the memristor is negligible. The array-level implementation depicted in Fig. 5a facilitates parallelism, leading to substantial time savings during training. For example, during $\phi _E$ = 1, eligibility is updated concurrently for all the elements in the array, followed by concurrent weight update at the end of the dataframe in $\phi _W$ = 1.

## Numerical modeling

**Fig. 6:**
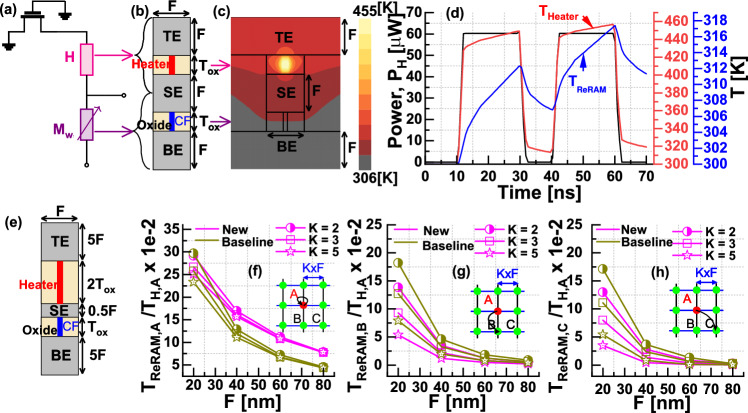
**a** 1T-1H-1M synapse unit cell. **b** Modeled geometry for electrothermal analysis. **c** Temperature contours calculated at *t* = 60ns for *F* = 60nm and overlaid on the modeled geometry, showing the thermal self-thermal coupling between the heater and ReRAM. **d** Transient temperature evolution depicting $\phi _{E}$ = 1 phase. **e** Modified geometry that improves self-thermal coupling and reduces the thermal crosstalk. A comparison of thermal coupling coefficients for the structure shown in (**b**) & (**e**) is shown in (**f**–**h**). **f** self-thermal coupling between heater and ReRAM at location *A*. Thermal crosstalk between the heater at location *A* and ReRAM at location *B* and *C* is shown in (**g**) & (**h**), respectively. The inset shows the schematic of the modeled 3$\times$3 crossbar array, where *K* denotes crossbar pitch.

The electrothermal effects are critical in the operation of the proposed synapse and are further investigated using the numerical model. Figure 6a shows the schematic of the 1*T*-1*H*-1*M* synapse, and the corresponding modeled geometry considered for electrothermal simulation is shown in Fig. 6b. The oxide thickness ($T_{\textrm{ox}}$) is assumed to be 30nm for both the heater and ReRAM (see Fig. 6b). All other dimensions are marked in minimum feature size (*F*). The time-dependent temperature profile within the device is obtained by solving the transient heat flow equation. More details on the numerical model are provided in the supplementary information (section 4).

Figure 6d illustrates the e-update phase, showing the transient temperature evolution at the heater and ReRAM in response to the dissipated power at the heater. Temperature contours calculated at $t = 60$ ns are overlaid on the modeled geometry and shown in Fig. 6c, highlighting the thermal coupling between the heater and ReRAM. Overall, Fig. 6c and d validate the capability to encode the “eligibility state” in the form of local temperature, as the local temperature of the ReRAM can be modulated by applying heating pulses at the heater.

We now examine potential sources of non-idealities that might influence the performance metrics of e-prop. For instance, the accumulated eligibility (e_Σ_) is expected to remain constant, even in the absence of activity (see Eq. 8 in the supplementary information (section 1)). However, in the proposed synapse, e_Σ_ decreases due to natural temperature decay in the absence of heating pulses (refer to Fig. 6d). Ideally, this suggests that the desired $\tau _\textrm{TH}$ should tend towards infinity. However, it will be evident in the next section that the desired value of $\tau _\textrm{TH}$ depends on the target application, and, in fact, this eligibility decay could be useful in certain cases. Additionally, it’s important to note that when the pulse width ($t_\textrm{PW}$) exceeds the device thermal time constant ($\tau _\textrm{TH}$), the ReRAM temperature reaches the steady state, impeding further e-updates. Thus, the $t_\textrm{PW}$ should be shorter than $\tau _\textrm{TH}$ during the $\phi _E = 1$ phase to update the eligibility state continuously.

Another important non-ideality is the thermal crosstalk during the $\phi _E=1$ phase. The unintentional rise in the local temperature of the neighboring synapses could result in an erroneous e-update. Therefore, we define the thermal crosstalk coefficient as the ratio of temperature rise at the adjacent synapses to temperature rise at the heater in response to heating pulse in $\phi _E$=1 phase. To mitigate the issue of thermal crosstalk, modifications are made to the device structure, as illustrated in Fig. 6e. These modifications include reducing the distance between the heater and ReRAM to enhance the desired self-thermal coupling and increasing the thickness of both the top and bottom electrodes to slow the propagation of heat flux toward neighboring devices, thereby reducing unintentional thermal crosstalk. Further, the thermal crosstalk coefficients for the structures shown in Fig. 6b, e are compared for different values *F* and *K*. Figure 6f shows that the new design increases the self-thermal coupling and reduces thermal crosstalk, as shown in Fig. 6g, h. The effects of these non-idealities, including eligibility decay and thermal crosstalk, are examined in detail in the benchmark simulations section.

## Benchmark simulations

### Case study #1: reinforcement learning in SNNs

**Fig. 7:**
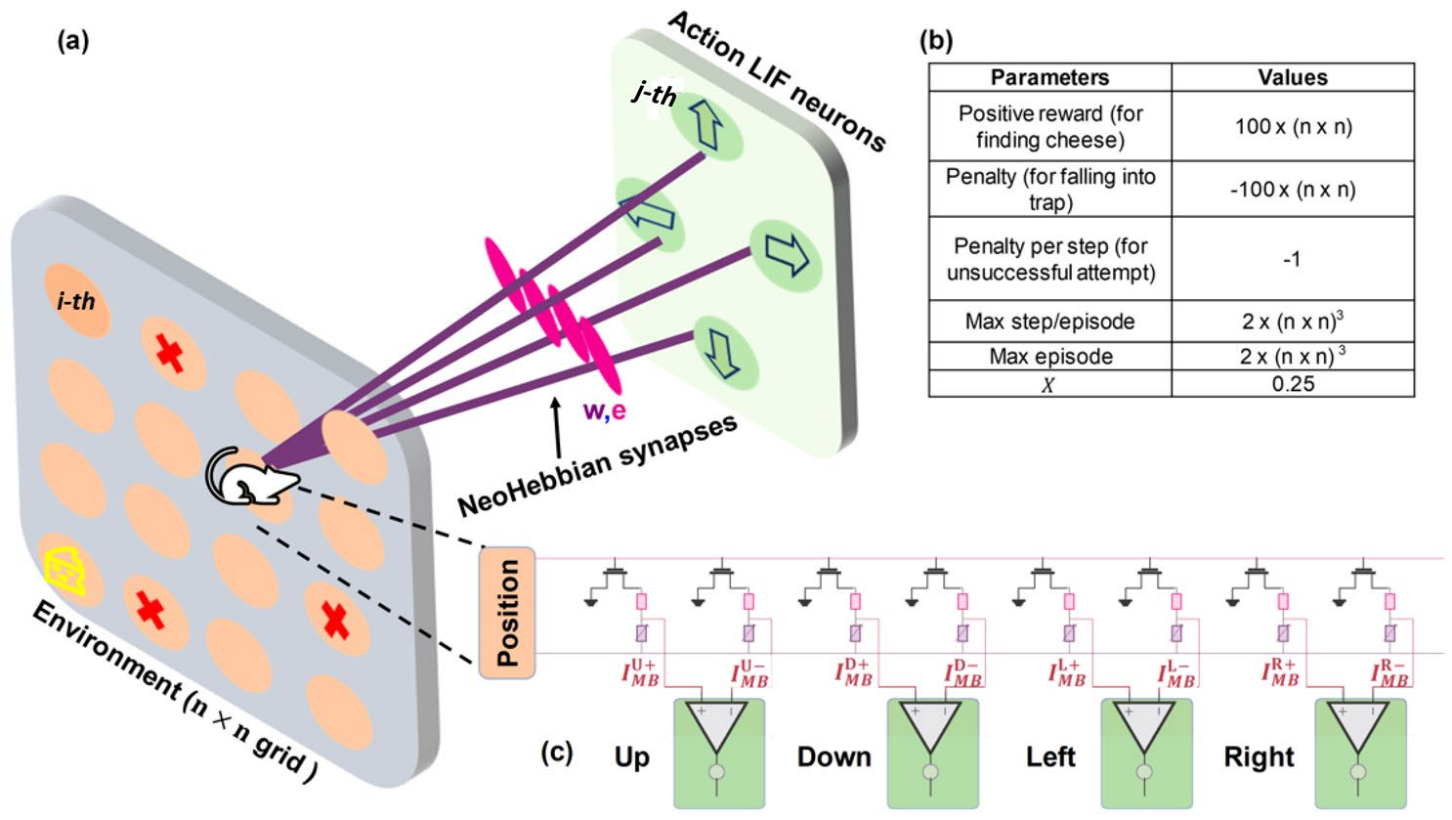
**a** Schematic of SNN used for illustrating reinforcement learning using neoHebbian synapse. Here, the agent, depicted as a mouse, must navigate through a $n \times n$ grid to locate cheese and avoid traps. The traps are shown using the red color cross. NeoHebbian synapse connects every *i*-th neuron in a $n \times n$ grid to every *j*-th neuron in the output layer (connection for only one *i*-th neuron is shown to avoid clutter). **b** Parameter values used in the simulations. **c** Schematic of array level implementation of the network shown in (a). Here $n \times n$ denotes the grid length.

This case study discusses the use of neoHebbian synapses in training SNNs for tasks related to reinforcement learning. Specifically, we explore a scenario where a virtual agent resembling a mouse navigates a maze in search of cheese while avoiding traps (see Fig. 7a). The maze is structured like a $n \times n$ grid, where the mouse’s current position defines its state. At each step, the agent, or mouse, is limited to a single action: moving in one of four directions “up”, “down”, “left”, or “right”. An episode in this context refers to a single run of the agent through the maze, from start to termination. Each episode begins with the agent randomly placed within the maze and terminates when the agent either finds cheese or encounters a trap. Following each episode, a new round commences from a randomly selected location within the maze. The agent is trained over multiple episodes, learning from past experiences to improve performance. The reward system is designed to maximize the agent’s chances of finding cheese, offering positive rewards for success and penalties for falling into traps. Additionally, each action made without finding cheese results in a minor penalty. Schematic of array level implementation of the network is shown in Fig. 7c. The respective parameters are summarized in the table shown in Fig. 7b.

In each episode, the agent navigates the grid by making decisions at every timestep. The assumed grid arrangement is akin to an input layer (environment position/state) where each location on the grid is connected to four Leaky-Integrate and Fire (LIF) neurons in the output layer (representing action) using thermal neoHebbian synapse as shown in Fig. 7a. The LIF neurons drive the agent’s decision-making process at each time step in the output layer. For example, suppose the LIF neuron corresponding to the action “up” direction in the output layer exhibits the highest membrane potential, which is influenced by both the current grid position of the mouse and the accumulated potential from the previous state. In that case, the mouse will move in the “up” direction. Further, homeostasis is applied on the most recent action (output LIF neuron with highest membrane potential) by decreasing the membrane potential by half.

During an episode, the eligibility value is updated at every time step according to the following procedure. Referring to Fig. 7a, suppose the agent is positioned at the *i*-th neuron in an $n \times n$ grid. If the membrane potential of the *j*-th action neuron is the highest, then the eligibility value for the synapse connecting the *i*-th position neuron with the *j*-th action neuron is increased. Therefore, the updated eligibility takes the form,

4 $$\begin{aligned} e_{i,j}(t) = e_{i,j}(t-1) + 1. \end{aligned}$$

Equation (4) reflects a Hebbian-like co-activation rule: whenever state *i* and action *j* are jointly active, the corresponding synapse accumulates eligibility. This discrete increment captures the idea that recently used state–action pairs are more likely to be credited when rewards arrive later. And the eligibility values of all other synapses undergo the leakage similar to works^53,54^,

5 $$\begin{aligned} e_{i,j}(t) = \gamma \; e_{i,j}(t-1). \end{aligned}$$

Here $\gamma$ is the discount factor, and it ranges between 0 to 1. Equation (5) implements exponential decay of eligibility for inactive synapses, which is consistent with eligibility traces in reinforcement learning and with the e-prop framework. This leakage prevents stale synapses from retaining undue credit and provides a biologically plausible fading memory of past activity. The synaptic weights are updated at the end of each episode in proportion to the accumulated rewards and eligibility value, as shown in the following equation.

6 $$\begin{aligned} \Delta W_{ij} = \bigg (\frac{1}{1+e^{-r}}-\chi \bigg )\cdot \sum ^{episode} e_{ij}. \end{aligned}$$

Here, *r* denotes the ratio of the accumulated rewards and penalties in an episode relative to the highest positive reward. Equation (6) combines the local eligibility with a global learning signal, consistent with the three-factor learning rule. The logistic term provides a bounded and normalized scaling of the reward relative to a baseline $\chi$ to ensure stable updates across different reward magnitudes. The weight update procedure ensures that both rewards and recent actions are considered during learning. The neuron membrane potential and eligibility values are reset to zero at the beginning of each episode. We scale the worst and best rewards proportional to grid length to standardize rewards across various grid sizes. This scaling approach ensures consistency in the reward magnitudes relative to the size of the grid (see Fig. 7b).

As discussed in earlier sections, the natural decay of temperature in the absence of heating pulses represents an important non-ideal aspect. This decay results in a reduction in accumulated eligibility, which is typically expected to remain constant until the weight update occurs (see eq.8 in the supplementary information section 1). However, in the context of the reinforcement learning scenario, this non-ideality facilitates the realization of the discount factor $\gamma$. This factor allows the agent to prioritize recent experiences while gradually diminishing the significance of older ones, which is crucial for effectively adapting to the changing challenges presented by the environment.

**Fig. 8:**
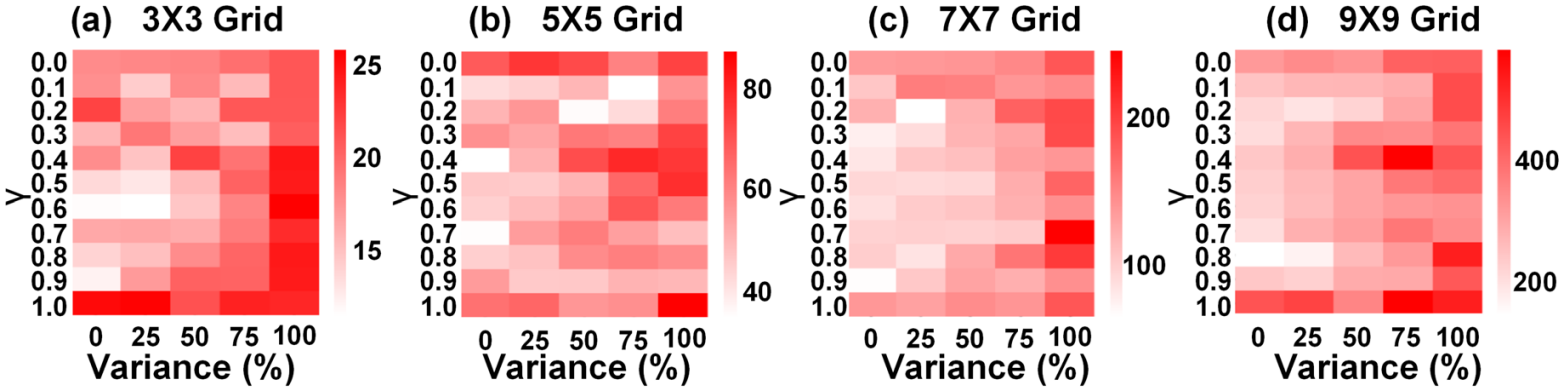
Heatmap compares the average number of episodes required to reach the learning benchmark across different grid sizes: **a** $3 \times 3$ grid, **b** $5 \times 5$ grid, **c** $7 \times 7$ grid, and **d** $10 \times 10$ obtained by considering the impact of temperature decay and memistor variability. Simulation results, which are the mean of 20 runs for each unique combination of $\gamma$ and variability in each grid size, are obtained using a pair of ReRAM devices in a differential configuration to implement a synapse, with each ReRAM device assumed to have 7-bit precision.

Benchmark simulations are performed on various $n\times n$ grids (*n* = 3, 5, 7, 10) to investigate the influence of temperature decay on the agent’s learning ability. In this context, “learning” refers to the agent’s ability to earn five consecutive positive rewards. Figure 8 compares the average number of episodes required to reach this learning benchmark across different grid sizes, considering the effects of temperature decay and ReRAM variability. For instance, in scenarios where $\gamma$ = 0, eligibility accumulation is null due to rapid temperature decay, while $\gamma$ = 1 signifies no reduction in accumulated eligibility owing to extremely slow temperature decay. When $\gamma$=0, it’s expected that there would be an increase in the average number of episodes required to reach the learning benchmark, as the agent doesn’t consider prior experiences while making decisions. Interestingly, it’s observed that the average number of episodes needed to reach the learning benchmark for $\gamma$ = 1 is also higher across all grid sizes, indicating that the agent struggles to achieve the benchmark if the temperature decay is extremely slow. This effect is particularly pronounced in larger grid sizes (n = 7, 10), where complexity and the number of possible paths are higher. The agent learns faster with optimal temperature decay, as indicated by the optimal $\gamma$ value in the heatmap, is considered. Therefore, the seemingly non-ideal effect of temperature decay proves beneficial in reinforcement learning, as it enables the agent to prioritize recent experiences and gradually diminishes the importance of past experiences. Moreover, increased ReRAM variability hampers the agent’s learning process, as evidenced by the rise in the average number of episodes needed to reach the learning benchmark with increasing variability. Details about the variability model used in our simulations are provided in the supplementary information section 5.

**Fig. 9:**
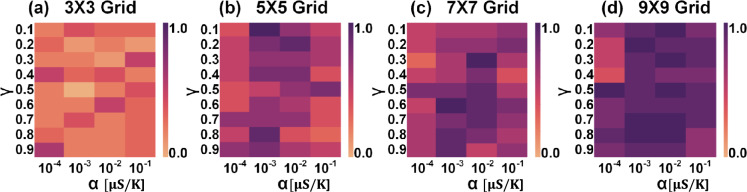
The heatmaps illustrate success ratios for a spiking neural network agent’s training across various grid sizes with $\gamma$ (y-axis) controls temperature decay, while $\alpha$ is varied along the x-axis. Lighter shades indicate lower success ratios. Simulation results, which are the mean of 20 runs for each unique combination of $\gamma$ and $\alpha$ in each grid size, are obtained using a pair of ReRAM devices in a differential configuration to implement a synapse, with each ReRAM device assumed to have 7-bit precision.

Next, with a fixed 50% variability, we analyze the impact of temperature-induced changes in ReRAM conductance, modeled as $W = W(1 + \alpha (T - T_\textrm{amb}))$, where $W$ is ReRAM conductance^55^. A lower $\alpha$ value is typically preferred in these applications. The heatmap in Fig. 9 shows the agent’s success ratio within a maximum number of episodes for each grid size. The success ratio represents the number of times the agent reached the learning benchmark for each unique combination of $\gamma$ and $\alpha$, divided by the maximum number of times the benchmark was reached. Figure 9 shows that the influence of $\alpha$ becomes less significant with increasing grid sizes, potentially due to the increased redundancy. This observation underscores the potential for efficient operation in dense arrays, a capability that will be further explored in a subsequent case study involving more complex networks. In practice, temperature-induced changes in ReRAM conductance, can be improved beyond by reducing $\alpha$ at the device level. Materials engineering strategies such as tailoring oxide stoichiometry to suppress vacancy diffusion, introducing dopants to stabilize conductive paths, or adding barrier/capping layers to reduce thermal activation have been shown to enhance stability. At the circuit and architecture level, approaches such as differential encoding to cancel common-mode drift, periodic refresh or re-training of synaptic weights, and redundancy across parallel devices can further mitigate conductance relaxation^51^.

**Fig. 10:**
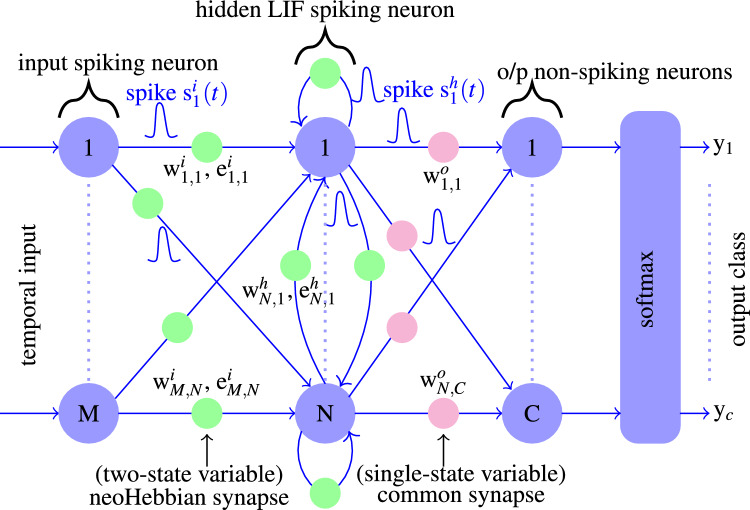
The schematic of the fully connected RSNN with one hidden layer. The input and hidden layers consist of spiking LIF neurons. NeoHebbian synapses connect the input layer with the hidden layer and recurrent connection within the hidden layer. The output readout and hidden layers are connected using (common) Hebbian synapses. $w^i_{ij}$, $w^h_{ij}$, $w^o_{ij}$ denote the synaptic weights in the input, hidden and output layer. $e_{ij}$ and *s*(*t*) denote the stored eligibility value and spikes from LIF neurons, respectively.

### Case study #2: RSNNs for phenome classification

In this case study, we investigate the performance of the thermal neoHebbian synapse in the TIMIT phoneme classification task. We employ the e-prop algorithm to conduct online training of recurrent spiking neural networks (RSNNs) featuring thermal neoHebbian synapses on the TIMIT dataset. TIMIT phoneme recognition serves as a standard measure for assessing the temporal processing capabilities of recurrent neural networks^56^. The dataset consists of acoustic speech signals from 630 speakers across eight dialect regions of the USA. The objective is to identify the spoken phoneme among 61 phonemes within each 10ms time frame.

**Fig. 11:**
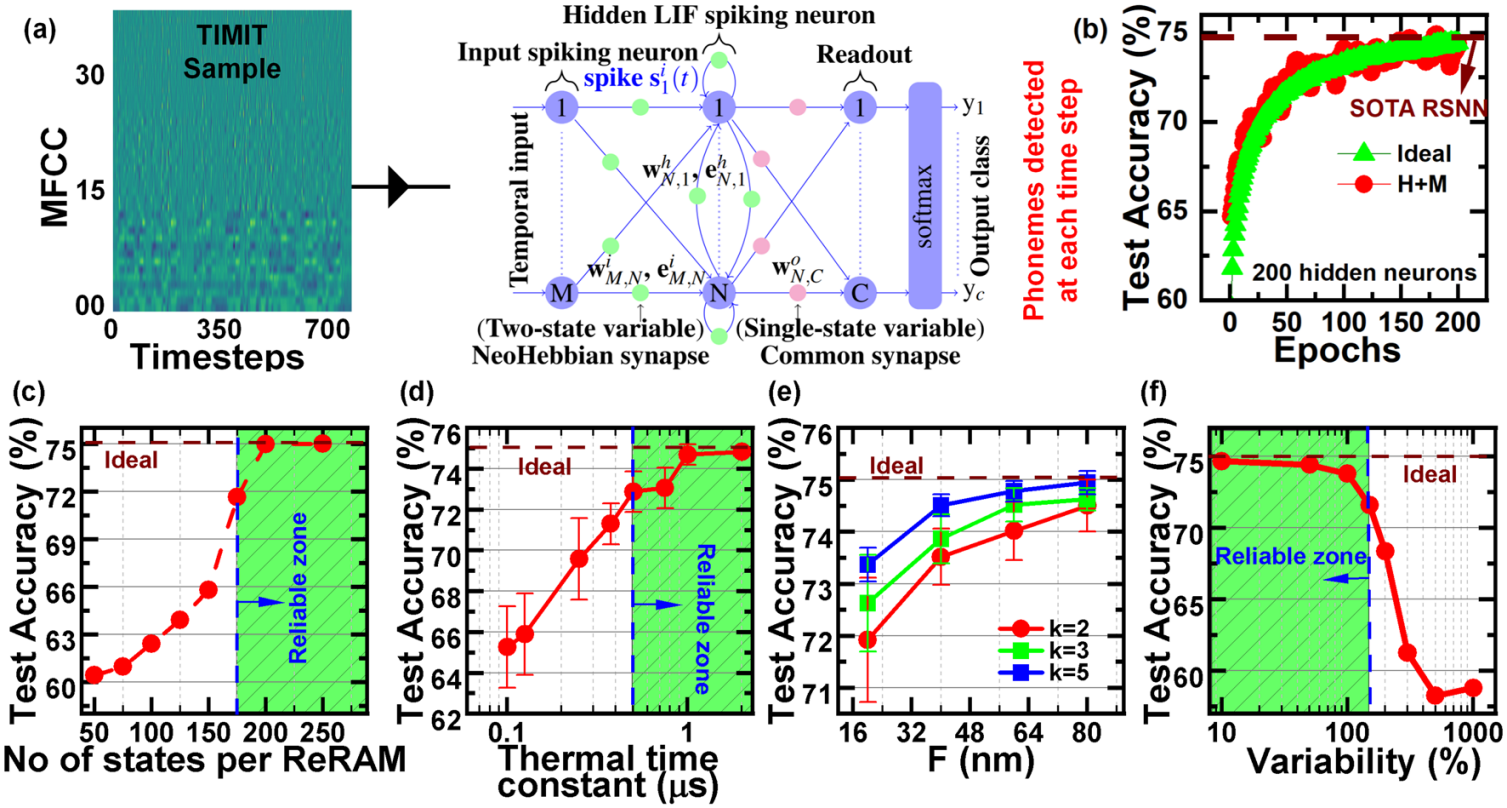
**a** A sample from the TIMIT data set applied to the input layer of the modeled RSNN used for the TIMIT phenome detection task. **b** Ideal (software modeled) synapse and proposed synapse test accuracy comparison (c,d,e,f) shows the test accuracy sensitivity towards various sources of non-idealities such as **c** Bit precision, **d** Thermal decay, **e** Thermal crosstalk, **f** Variability.

The schematic representation of the modeled RSNN network used in this study is illustrated in Fig. 10, comprising 39 input neurons, one hidden layer with 200 LIF neurons, and an output layer consisting of 61 neurons, operating over an average of 700 time steps per sample during inference. The input data is encoded following the procedure outlined in^36^, and one sample input data is shown in Fig. 11a. The LIF spiking neurons in the input layers are connected to the hidden layer LIF neurons via neoHebbian synapses. Hidden layer LIF neurons are recurrently connected to themselves and other neurons by neoHebbian synapses. An output (readout) layer of non-spiking neurons is connected to the hidden layer with common (Hebbian) synapses. Spikes coming from the input layer ($s^{i}(t)$) and recurrent connections ($s^{h}(t)$) updates the membrane potential of the hidden layer neurons. The training operation is performed as follows. During the e-update phase, the eligibility contribution is computed at each time step and accumulated at synapse during the presentation of *U*-step long input dataframe as follows,

7 $$\begin{aligned} e_{\Sigma } = \sum _{t=1}^U e_{ij}(t), \text { where } e_{ij}(t) = f_{i}(t) \times \psi _{j}(t). \end{aligned}$$

Here, *f*(*t*) and $\psi (t)$ signals are provided from pre-synaptic and post-synaptic neurons, respectively. The network loss is calculated at the non-spiking output neurons, and batch-mode stochastic gradient descent is used to update the output layer weights ($w^o$). The neoHebbian synapse ($w^{i / h}$) are updated according to

8 $$\begin{aligned} \Delta w^{i / h} = \eta e_{\Sigma }. \end{aligned}$$

Here, parameter $\eta$ denotes the learning rate. The values of $e_{\Sigma }$, *f*(*t*), and $\psi (t)$ are set to zero at $t=0$, i.e., before the presentation of a new training dataframe. Note that updates to the readout weights ($w^o$) and input/recurrent neoHebbian weights ($w^{i / h}$) occur exclusively at the end of each dataframe. supplementary information section 1 provides more details on the key equations used in this case study.

Figure 11b compares the training accuracy obtained using ideal (software-modeled) synapses and the proposed neoHebbian synapses. The proposed synapses perform comparably to ideal synapses, assuming floating-point precision. We then investigated the dependence of test accuracy on synapse bit precision, as shown in Fig. 11c. Our study demonstrates that a minimum of 200 states per ReRAM (approximately 8-bit precision) is required to ensure that the degradation in test accuracy is less than 3%.

Two significant sources of non-idealities specific to the thermal neoHebbian synapse include thermal crosstalk and temperature decay. It is noted that test accuracy increases with an increase in $\tau _\textrm{TH}$ and saturates for $\tau _\textrm{TH}$ values exceeding 1$\upmu s$, as depicted in Fig. 11d. The choice of materials, device dimensions, and crossbar size dictates the $\tau _\textrm{TH}$ value, and achieving $\tau _\textrm{TH} \approx 1 \upmu s$ is feasible with practical crossbar arrays^44,57^. Thermal crosstalk becomes a critical factor with higher device density, i.e., as minimum feature size (*F*) and crossbar pitch (*K*) decrease. To evaluate the impact of thermal crosstalk, synapse locations are considered from $N\times M$ and $M\times M$ crossbar implementations for the input and recurrent layers, respectively. The data provided in Fig. 6f–h is used to obtain the thermal coupling coefficient. Figure 11e demonstrates that despite notable scaling in *F* and *K*, the reduction in test accuracy is approximately 3%. Both transistor scaling and thermal crosstalk are critical in determining the scaling potential of thermal synapses. Lastly, Fig. 11f shows the test accuracy’s dependence on memristor variability, showing a decrease of around 1% for variations up to 100%. It is shown that increasing the network size results in an improvement in test accuracy. Thus, we attribute the network resilience to various non-idealities to the inherent redundancy in the baseline network^36^ and the implementation of hardware-aware training techniques^48^. Details on the memristor variability model, its impact on network performance, and the effects of increased ambient temperature on test accuracy are provided in supplementary information Section 5.

Table 1 compares key metrics of the proposed synapse against the prior works. Per synapse area is determined assuming 1T-1R unit cell configuration, where the heater element is integrated above the ReRAM, resulting in no additional area overhead. The ReRAM cross-sectional area is assumed to be $250\times 250\, \hbox {nm}^2$, with 200 nm spacing between metal lines, giving an estimated cell area of $\hbox {450F}^2$. Based on 65nm technology for the access transistors, the total cell area is $1.9\, \upmu \hbox {m}^2$. We note that the choice of 65nm technology for access transistor is driven by our fabricated ReRAM’s switching voltages, switching currents, and conductance range^10^. However, further reductions unit cell area are possible by decreasing the ReRAM cell area, switching voltages and currents^58^. The total energy of the proposed synapse is estimated assuming a 10 ns spike integration time and a write voltage ($V_\textrm{w}$) of 1.7 V. The energy per timestep for inference and weight update is on the order of $\sim V_\textrm{w}^{2} G_\textrm{w} t_\textrm{PW} \approx$ a few fJ (e.g., $\sim$4 fJ/timestep for $V_\textrm{read}=0.1$ V, $t_\textrm{PW}=10$ ns, and $V_\textrm{write}=1.7$ V). In addition, the eligibility update phase in our design involves activation of a local heater; assuming a heater resistance of 500 $\Omega$ and a 10 ns activation pulse, the corresponding energy consumption is estimated to be $\sim$4.5 pJ per timestep. In conclusion, the proposed synapse offers competitive advantages in terms of both area and energy efficiency.

**Table 1:** Comparison of the proposed synapse with prior works. $^{\$}$Calculated assuming SRAM cell area of $\hbox {150F}^{2}$ in 28 nm technology and 8-bit weights. $^{\Lambda }$Calculated assuming 2T1R unit cell and 14nm technology for the access transistor as per^14^. $^\Upsilon$Based on fabricated $\hbox {Ag/GeSe}_{3}$/Ag device dimensions as per^60^. $^{+}$Based on 65nm technology for access transistor and cell area of $\hbox {450F}^2$. $^!$Write energy calculated ($\approx I^2 t_{pw} / G$) assuming I$_{prog}$ $\sim$ 100 $\upmu$A, $G \sim$ 1 $\upmu$S, and $t_{pw} \sim$ 100ns, as per the parameters mentioned in^50^. &Learning energy reported at V= 0.5V^59^. $^*$Only coupling weights are PCM-based; eligibility computations are performed using a high-precision unit. Write energy is estimated roughly according to the values provided in^49^: I$_{prog}$ = 700$\,\upmu$A, $t_{pw}$ = 600ns, $G = 10\, \upmu$S. $^\#$Learning energy dominated by optical power^60^. $^\otimes$ Limited by PCM device resistance drift rate. $^\boxtimes$Limited by Von-neumann style sequential computing. $^\triangle$Limited by Ag conductive filament relaxation dynamics. $^\Omega$Limited by thermal time constant.

	Coupling weights	Eligibility	Per synapse area	Energy per timestep	Eligibility decay time constant	Maturity
Y. Demirağ et al.^50^	PCM conductance	PCM drift	$12 \times 12 \, \upmu \textrm{m}^2$	$\sim 1 \, \textrm{nJ}^{!}$	$\sim \textrm{s}^{\otimes }$	+
C. Frenkel et al.^59^	CMOS	CMOS	$1200F^{2 \, \$}$ (0.94 μm^2^)	$1.5-178 \, \textrm{nJ}^{ \& }$	$\sim \textrm{ms}^{\boxtimes }$	++
T. Bohnstingl et al.^49^	PCM conductance	CMOS	$5221F^{2 \, \Lambda }$ (1.02 μm^2^)	$29 \, \textrm{nJ}^{*}$	$\sim \textrm{ms}^{\boxtimes }$	+
S. G. Sarwat et al.^60^	PCM conductance	Optical response of PCM	$5 \times 4.8 \, \upmu \textrm{m}^2 \, \Upsilon$	$\sim \textrm{mJ}^{\#}$	$\sim \mathrm {ns{-}ms}^{\triangle }$	-
This work	ReRAM conductance	ReRAM local temperature	$450F^2$ (1.9 μm^2^)	$5 \, \textrm{pJ}$	$\sim \mathrm {ns{-}\upmu s}^{\Omega }$	-

## Discussion & summary

The proposed thermal neoHebbian synapse leverages both thermal and electrical effects in computation, forming a multi-physics computing unit. This approach offers several advantages over conventional computation methods. For example, conventional computing units are burdened with converting all signals into the electrical domain, including voltages, currents, and conductances, neglecting other forms of information generated during network operation. By harnessing both electrical and thermal effects in computation, we can maximize the utilization of information derived from network activity, potentially leading to significant improvements^61^.

This approach has gained increasing attention in recent years^44,62–66^. For instance, Kim et al.^65^ experimentally demonstrated that the dynamic evolution of internal state variables, particularly temperature, enables ReRAM to mimic $\hbox {Ca}^{2+}$-like dynamics, facilitating the native encoding of temporal information and synaptic weight regulation. They showed that these internal dynamics can be exploited to implement spike-timing-dependent plasticity (STDP) using simple, non-overlapping pulses. Building on this, Yoo et al.^44^ proposed material and structural modifications to enhance internal temperature dynamics, validating the concept through the application of STDP-trained spiking neural networks for temporal correlation detection tasks. Another study^66^ explored the use of thermal crosstalk in neuromorphic computing, proposing its potential for future applications. In related work, it shows that the thermal crosstalk-driven spatiotemporal communication in multiple Mott neurons achieves energy efficiency several orders of magnitude greater than state-of-the-art digital processors^62^. Similarly, Kumar et al.^63^ leveraged thermal dynamics to demonstrate 15 distinct neuronal behaviors using nanoscale third-order circuit elements, showing promise for the development of highly efficient neuromorphic hardware.

While multi-physics computing units, particularly those involving temperature, offer significant advantages, they also present several practical challenges. Unlike measurable variables such as current or voltage, temperature is a hidden variable, making direct measurement and control difficult. Furthermore, elevated temperatures can accelerate device degradation and lead to various reliability issues^67,68^. Although this work and several other works^44,62–66^ demonstrated a method of exploiting thermal effects for computation, significant challenges remain for future real-world applications.

In addition, heat dissipation is an unavoidable byproduct of electronic system operation, and it is increasingly pronounced as devices continue to shrink in size^69^. While efforts to reduce power dissipation remain a priority, exploring innovative approaches to harness electro-thermal effects could unlock new possibilities. For example, such approaches could drive advancements in novel materials with tailored thermal properties, where electronic and thermal behaviors can be independently controlled. Moreover, the development of nanoscale devices capable of regulating heat flow, such as thermal diodes or thermal transistors, presents promising directions for future research^70,71^.

In summary, we have proposed and experimentally validated ReRAM-based neo-Hebbian synapses. The performance improvements provided by these synapses were evaluated through two representative applications based on the scalable e-prop learning algorithm. Our findings demonstrate that the proposed thermal neo-Hebbian synapses significantly reduce both time-to-solution and energy-to-solution. This underscores their potential for facilitating fast, scalable, online, and robust learning in neuromorphic hardware.

## Data Availability

The data that support the findings of this study are available from the corresponding author upon reasonable request.

## References

[1] Mead, C. Neuromorphic electronic systems. *Proc. IEEE* **78**, 1629–1636. 10.1109/5.58356 (1990).

[2] Mead, C. Author correction: How we created neuromorphic engineering. *Nat. Electron.* **3**, 579 (2020).

[3] Davies, M. et al. Advancing neuromorphic computing with loihi: A survey of results and outlook. *Proc. IEEE* **109**, 911–934. 10.1109/JPROC.2021.3067593 (2021).

[4] Schuman, C. D. et al. Opportunities for neuromorphic computing algorithms and applications. *Nat. Comput. Sci.* **2**, 10–19 (2022). 38177712 10.1038/s43588-021-00184-y

[5] Marković, D., Mizrahi, A., Querlioz, D. & Grollier, J. Physics for neuromorphic computing. *Nat. Rev. Phys.* **2**, 499–510 (2020).

[6] Mehonic, A. et al. Memristors-from in-memory computing, deep learning acceleration, and spiking neural networks to the future of neuromorphic and bio-inspired computing. *Adv. Intell. Syst.* **2**, 2000085. 10.1002/aisy.202000085 (2020).

[7] Roy, K., Jaiswal, A. & Panda, P. Towards spike-based machine intelligence with neuromorphic computing. *Nature* **575**, 607–617 (2019). 31776490 10.1038/s41586-019-1677-2

[8] Burr, G. W. et al. Neuromorphic computing using non-volatile memory. *Adv. Phys.: X* **2**, 89–124 (2017).

[9] Upadhyay, N. K. et al. Emerging memory devices for neuromorphic computing. *Adv. Mater. Technol.* **4**, 1800589 (2019).

[10] Kim, H., Mahmoodi, M. R., Nili, H. & Strukov, D. B. 4k-memristor analog-grade passive crossbar circuit. *Nat. Commun.* **12**, 5198 (2021). 34465783 10.1038/s41467-021-25455-0 PMC8408216

[11] Yang, J. J., Strukov, D. B. & Stewart, D. R. Memristive devices for computing. *Nat. Nanotechnol.* **8**, 13–24 (2013). 23269430 10.1038/nnano.2012.240

[12] Ielmini, D. & Wong, H.-S.P. In-memory computing with resistive switching devices. *Nat. Electron.* **1**, 333–343 (2018).

[13] Song, M.-K. et al. Recent advances and future prospects for memristive materials, devices, and systems. *ACS Nano* **17**, 11994–12039. 10.1021/acsnano.3c03505 (2023). 37382380 10.1021/acsnano.3c03505

[14] Gallo, M. L. et al. A 64-core mixed-signal in-memory compute chip based on phase-change memory for deep neural network inference. *Nat. Electron.* **6**, 680–693 (2022).

[15] Ambrogio, S. et al. An analog-ai chip for energy-efficient speech recognition and transcription. *Nature* **620**, 768–775 (2023). 37612392 10.1038/s41586-023-06337-5 PMC10447234

[16] Sebastian, A. Analog in memory computing for deep learning inference. In *IEEE International Electron Devices Meeting*.

[17] Huang, Y. et al. Memristor-based hardware accelerators for artificial intelligence. *Nat. Rev. Electr. Eng.* **1**(5), 286–99 (2024).

[18] Natschläger, T. & Maass, W. Information dynamics and emergent computation in recurrent circuits of spiking neurons. In *Advances in Neural Information Processing Systems*, vol. 16 (MIT Press, 2003).

[19] Li, G. et al. Brain inspired computing: A systematic survey and future trends. *TechRxiv* (2023).

[20] Mehonic, A. & Kenyon, A. Brain-inspired computing needs a master plan. *Nature*10.1038/s41586-021-04362-w (2022). 35418630 10.1038/s41586-021-04362-w

[21] Ganguly, C. et al. Spike frequency adaptation: bridging neural models and neuromorphic applications. *Commun. Eng.* **3**, 22. 10.1038/s44172-024-00165-9 (2024).

[22] Caporale, N. & Dan, Y. Spike timing-dependent plasticity: a Hebbian learning rule. *Ann. Rev. Neurosci.* **31**, 25–46 (2008). 18275283 10.1146/annurev.neuro.31.060407.125639

[23] Bi, G. & Ming Poo, M. Synaptic modifications in cultured hippocampal neurons: Dependence on spike timing, synaptic strength, and postsynaptic cell type. *J. Neurosci.* **18**, 10464–10472 (1998). 9852584 10.1523/JNEUROSCI.18-24-10464.1998 PMC6793365

[24] Prezioso, M. et al. Spike-timing-dependent plasticity learning of coincidence detection with passively integrated memristive circuits. *Nat. Commun.* **9**, 5311 (2018). 30552327 10.1038/s41467-018-07757-y PMC6294012

[25] Serrano-Gotarredona, T., Masquelier, T., Prodromakis, T., Indiveri, G. & Linares-Barranco, B. STDP and STDP variations with memristors for spiking neuromorphic learning systems. *Front. Neurosci.*10.3389/fnins.2013.00002 (2013). 23423540 10.3389/fnins.2013.00002 PMC3575074

[26] Milo, V. et al. Resistive switching synapses for unsupervised learning in feed-forward and recurrent neural networks. In *2018 IEEE International Symposium on Circuits and Systems (ISCAS)*, 1–5. 10.1109/ISCAS.2018.8351824. ISSN: 2379-447X.

[27] Gupta, A. & Saurabh, S. On-chip unsupervised learning using STDP in a spiking neural network. *IEEE Trans. Nanotechnol.* **22**, 365–376. 10.1109/TNANO.2023.3293011 (2023).

[28] Prezioso, M. et al. Spike-timing-dependent plasticity learning of coincidence detection with passively integrated memristive circuits. *Nat. Commun.* **9**, 5311. 10.1038/s41467-018-07757-y (2018). 30552327 10.1038/s41467-018-07757-y PMC6294012

[29] DeBole, M. V. et al. Truenorth: Accelerating from zero to 64 million neurons in 10 years. *Computer* **52**, 20–29 (2019).

[30] Orchard, G. et al. Efficient neuromorphic signal processing with loihi 2. In *2021 IEEE Workshop on Signal Processing Systems (SiPS)*, 254–259 (IEEE, 2021).

[31] Benjamin, B. V. et al. Neurogrid: A mixed-analog-digital multichip system for large-scale neural simulations. *Proc. IEEE* **102**, 699–716. 10.1109/JPROC.2014.2313565 (2014).

[32] Gonzalez, H. A. et al. Spinnaker2: A large-scale neuromorphic system for event-based and asynchronous machine learning. Preprint arXiv:2401.04491 (2024).

[33] Deng, L. et al. Tianjic: A unified and scalable chip bridging spike-based and continuous neural computation. *IEEE J. Solid-State Circuits* **55**, 2228–2246. 10.1109/JSSC.2020.2970709 (2020).

[34] Qiao, N. et al. A reconfigurable on-line learning spiking neuromorphic processor comprising 256 neurons and 128k synapses. *Front. Neurosci.* **9**, 123487 (2015). 10.3389/fnins.2015.00141 PMC4413675 25972778

[35] Gerstner, W., Lehmann, M. P., Liakoni, V., Corneil, D. S. & Brea, J. Eligibility traces and plasticity on behavioral time scales: Experimental support of Neohebbian three-factor learning rules. *Front. Neural Circuits* **12**, 53 (2018). 30108488 10.3389/fncir.2018.00053 PMC6079224

[36] Bellec, G. et al. A solution to the learning dilemma for recurrent networks of spiking neurons. *Nat. Commun.* **11**, 3625 (2019). 10.1038/s41467-020-17236-y PMC7367848 32681001

[37] Bellec, G., Salaj, D., Subramoney, A., Legenstein, R. A. & Maass, W. Long short-term memory and learning-to-learn in networks of spiking neurons. In *Neural Information Processing Systems* (2018).

[38] Werbos, P. Backpropagation through time: what it does and how to do it. *Proc. IEEE* **78**, 1550–1560. 10.1109/5.58337 (1990).

[39] Lillicrap, T. P. & Santoro, A. Backpropagation through time and the brain. *Curr. Opin. Neurobiol.* **55**, 82–89. 10.1016/j.conb.2019.01.011 (2019). 30851654 10.1016/j.conb.2019.01.011

[40] Marschall, O., Cho, K. & Savin, C. A unified framework of online learning algorithms for training recurrent neural networks. *J. Mach. Learn. Res.* **21**, 5320–5353 (2020).

[41] Fong, S. W., Neumann, C. M. & Wong, H.-S.P. Phase-change memory-towards a storage-class memory. *IEEE Trans. Electron Dev.* **64**, 4374–4385. 10.1109/TED.2017.2746342 (2017).

[42] Burr, G. W. et al. Recent progress in phase-change memory technology. *IEEE J. Emerg. Sel. Top. Circuits Syst.* **6**, 146–162 (2016).

[43] Ehrmann, A., Błachowicz, T., Ehrmann, G. & Grethe, T. Recent developments in phase-change memory. *Appl. Res.*10.1002/appl.202200024 (2022).

[44] Yoo, S., Wu, Y., Park, Y. & Lu, W. D. Tuning resistive switching behavior by controlling internal ionic dynamics for biorealistic implementation of synaptic plasticity. *Adv. Electron. Mater.* **8**, 2101025 (2022).

[45] Alibart, F., Gao, L., Hoskins, B. D. & Strukov, D. B. High precision tuning of state for memristive devices by adaptable variation-tolerant algorithm. *Nanotechnology* **23**, 075201 (2012). 22260949 10.1088/0957-4484/23/7/075201

[46] Larentis, S., Nardi, F., Balatti, S., Gilmer, D. C. & Ielmini, D. Resistive switching by voltage-driven ion migration in bipolar rram-part ii: Modeling. *IEEE Trans. Electron Devices* **59**, 2468–2475. 10.1109/TED.2012.2202320 (2012).

[47] Nardi, F., Larentis, S., Balatti, S., Gilmer, D. C. & Ielmini, D. Resistive switching by voltage-driven ion migration in bipolar rram-part i: Experimental study. *IEEE Trans. Electron Devices* **59**, 2461–2467. 10.1109/TED.2012.2202319 (2012).

[48] Bhattacharya, T., Bezugam, S., Pande, S., Wlazlak, E. & Strukov, D. Reram-based neohebbian synapses for faster training-time-to-accuracy neuromorphic hardware. In *2023 International Electron Devices Meeting (IEDM)*, 1–4. 10.1109/IEDM45741.2023.10413797 (2023).

[49] Bohnstingl, T. et al. Biologically-inspired training of spiking recurrent neural networks with neuromorphic hardware. In *2022 IEEE 4th International Conference on Artificial Intelligence Circuits and Systems (AICAS)*, 218–221. 10.1109/AICAS54282.2022.9869963 (2022).

[50] Demirağ, Y. et al. Pcm-trace: Scalable synaptic eligibility traces with resistivity drift of phase-change materials. In *2021 IEEE International Symposium on Circuits and Systems (ISCAS)*, 1–5. 10.1109/ISCAS51556.2021.9401446 (2021).

[51] Boybat, I. et al. Neuromorphic computing with multi-memristive synapses. *Nat. Commun.* **9**, 2514 (2017). 10.1038/s41467-018-04933-y PMC6023896 29955057

[52] Mahmoodi, M. R., Vincent, A. F., Nili, H. & Strukov, D. B. Intrinsic bounds for computing precision in memristor-based vector-by-matrix multipliers. *IEEE Trans. Nanotechnol.* **19**, 429–435. 10.1109/TNANO.2020.2992493 (2020).

[53] Espino, H., Bain, R. & Krichmar, J. L. A rapid adapting and continual learning spiking neural network path planning algorithm for mobile robots. Preprint arXiv:2404.15524 (2024).

[54] Galloni, A. R. et al. Neuromorphic one-shot learning utilizing a phase-transition material. *Proc. Natl. Acad. Sci.* **121**, e2318362121 (2024). 38630718 10.1073/pnas.2318362121 PMC11047090

[55] Nili, H. et al. Comprehensive compact phenomenological modeling of integrated metal-oxide memristors. *IEEE Trans. Nanotechnol.* **19**, 344–349. 10.1109/TNANO.2020.2982128 (2020).

[56] Garofolo, J. S. Timit acoustic phonetic continuous speech corpus. *Linguistic Data Consortium, 1993* (1993).

[57] Sun, P. et al. Thermal crosstalk in 3-dimensional rram crossbar array. *Sci. Rep.*10.1038/srep13504 (2015). 26310537 10.1038/srep13504 PMC4550907

[58] Golonzka, O. et al. Non-volatile rram embedded into 22ffl finfet technology. In *2019 Symposium on VLSI Technology*, T230–T231. 10.23919/VLSIT.2019.8776570 (2019).

[59] Frenkel, C. & Indiveri, G. Reckon: A 28nm sub-mm2 task-agnostic spiking recurrent neural network processor enabling on-chip learning over second-long timescales. In *2022 IEEE International Solid-State Circuits Conference (ISSCC)*, vol. 65, 1–3. 10.1109/ISSCC42614.2022.9731734 (2022).

[60] Sarwat, S. G., Moraitis, T., Wright, C. D. & Bhaskaran, H. Chalcogenide optomemristors for multi-factor neuromorphic computation. *Nat. Commun.* **13**, 2247 (2021). 10.1038/s41467-022-29870-9 PMC9042832 35474061

[61] Patel, R. K. & Ramanathan, S. Heat-assisted neuromorphic computing. *Nat. Mater.* **23**(9), 1157–8 (2024). 38890485 10.1038/s41563-024-01928-7

[62] Kim, K. M. et al. Computing with heat using biocompatible mott neurons. Research Square. 10.21203/rs.3.rs-3134569/v1 (2023)

[63] Kumar, S., Williams, R. S. & Wang, Z. Third-order nanocircuit elements for neuromorphic engineering. *Nature* **585**, 518–523 (2020). 32968256 10.1038/s41586-020-2735-5

[64] Li, R. *et al.* Thermal-induced multi-state memristors for neuromorphic engineering. In *2023 IEEE International Symposium on Circuits and Systems (ISCAS)*, 1–5. 10.1109/ISCAS46773.2023.10182122 (2023).

[65] Kim, S. et al. Experimental demonstration of a second-order memristor and its ability to biorealistically implement synaptic plasticity. *Nano Lett.* **15**(3), 2203–11 (2015). 25710872 10.1021/acs.nanolett.5b00697

[66] Schön, D. & Menzel, S. Spatio-temporal correlations in memristive crossbar arrays due to thermal effects. *Adv. Funct. Mater.*10.1002/adfm.202213943 (2023).

[67] Chang, Y.-F., Karpov, I. & et al., H. Embedded emerging memory technologies for neuromorphic computing: temperature instability and reliability. In *2021 IEEE International Reliability Physics Symposium (IRPS)*, 1–5. 10.1109/IRPS46558.2021.9405120 (2021).

[68] Torres, F., Basaran, A. & Schuller, I. Thermal management in neuromorphic materials, devices, and networks. *Adv. Mater.*10.1002/adma.202205098 (2023). 36067752 10.1002/adma.202205098

[69] Salahuddin, S. S., Ni, K. & Datta, S. The era of hyper-scaling in electronics. *Nat. Electron.* **1**, 442–450 (2018).

[70] Yang, Q. et al. Solid-state electrochemical thermal transistors. *Adv. Funct. Mater.*10.1002/adfm.202214939 (2023).39281808

[71] Wei, D. et al. Electric-controlled tunable thermal switch based on Janus monolayer Mosse. *npj Comput. Mater.* **8**, 1–7 (2022).

